# WildMinnie: compression of software-defined networking (SDN) rules with wildcard patterns

**DOI:** 10.7717/peerj-cs.809

**Published:** 2022-02-08

**Authors:** Hamed Khanmirza

**Affiliations:** Department of Computer Engineering, K. N. Toosi University of Technology, Tehran, Iran

**Keywords:** Software-defined networking, OpenFlow, Rule compression, Data center networks

## Abstract

Software-defined networking (SDN) enables fast service innovations through network programmability. In SDN, a logically centralized controller compiles a set of policies into the network-level rules. These rules are inserted in the TCAM memory of SDN-enabled switches enabling high-speed matching and forwarding of packets. Unfortunately, TCAMs are available in limited capacities and fall short of accommodating all intended rules, especially in networks with large distinct flows like datacenters. Rule compression is a technique that reduces the number of rules by aggregating them with some similarity factors. This paper introduces WildMinnie, a new rule compression method that aggregates rules based on their common address non-prefix wildcards derived from a group of rules with the same output port number. We explore rule conflict issues and provide solutions to resolve them. We demonstrate the capability of WildMinnie in various datacenter topologies with traffics having different diversity of source-destination addresses and show that WildMinnie outperforms the best-known compression method by 20%, on average.

## Introduction

**Figure 1:**
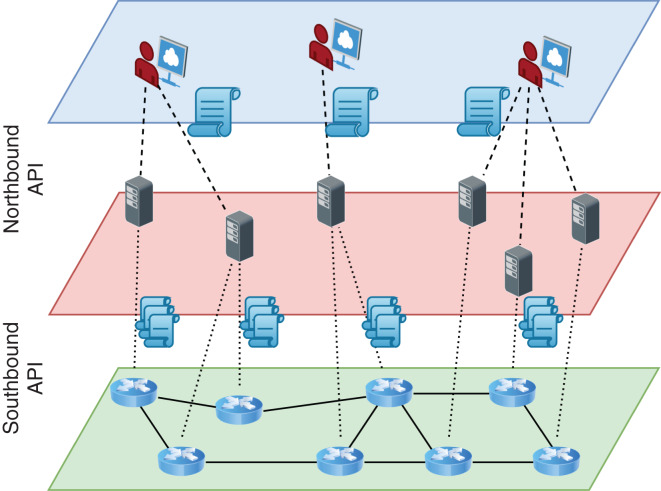
Logical and layered view of an SDN architecture.

Software-Defined Networking (SDN) enabled a significant shift from distributed autonomous network elements to centrally programmable elements. Such a shift facilitated better monitoring and faster innovation of new services (Feamster, Rexford & Zegura, 2014; Kreutz et al., 2015). In SDN, switches are still the main elements of the network data plane, but they work passively. The controller, a logically central software-based element, gets high-level policies from the administrator of a network and compiles them into the network-level rules (Fig. 1). These low-level rules are installed in switches using special communication protocols compliant with southbound API such as OpenFlow (McKeown et al., 2008). The southbound API provides a clean abstraction between the different implementations of network controllers and the technology employed in the data plane and makes possible independent development of both sides.

SDN switches usually use TCAM memories to speed up the time-consuming operation of matching rules with packet headers. Unfortunately, these memory types are costly and energy-hungry and come in limited capacities of a few thousands or tens of thousands (Stephens et al., 2012). This limitation causes switches not to keep rules of all passing flows, especially in networks with a high count of distinct flows.

To come up with this issue, earlier SDN architectures (Gude et al., 2008; Casado et al., 2009; Erickson, 2013; FloodLight, 2014) keep recently used rules in TCAM and force switches to contact the controller upon receiving the first packet of an unknown flow. Such a reactive behavior creates initial flow set-up delays and imposes a significant overhead on the controller in a rather dynamic network.

Meanwhile, some researchers such as Yu et al. (2011) believe that the routing task should be accomplished collectively by switches in the data plane to make the network scalable. They use sophisticated rule placement algorithms to distribute rules in the data plane such that all packets, eventually, find their path toward their intended destinations without communicating with the controller (Casado et al., 2009; Kang et al., 2013; Nguyen et al., 2015a; Huang et al., 2015; Ashraf, 2016; Kosugiyama et al., 2017; Galan-Jimenez, Polverini & Cianfrani, 2018; Zhang et al., 2018; Bera, Misra & Jamalipour, 2019; Zhao et al., 2020). The rule distribution and placement in these works are performed through innovative routing methods.

Rule compression is another line of solutions in which researchers, using various techniques, try to reduce the number of effective rules in each switch (Giroire, Moulierac & Phan, 2014). Rule compression may be used as a complement to other solutions to provide more space in rule tables and is not necessarily tied to any specific routing mechanism. Among compression approaches, Minnie (Rifai et al., 2017) introduces a powerful and more general multi-field aggregation method and achieves a 70–99% compression ratio in all-to-all scenarios, reportedly. In a nutshell, Minnie groups rules using the exact values of one field^1^ 1Theoretically, it is not important which field is used for compression. However, authors have only used source or destination address fields in their experiments.. Then, the most-used output ports within each group and among all groups are found. Finally, all rules with the globally most-used output port are substituted with the default rule. Other rules with the group-wide mostly used output ports are replaced with an applicable wildcard rule.

This paper proposes WildMinnie compression that works principally similar to Minnie; however, it groups rules with their common wildcard values instead of their exact values. In WildMinnie, at first, we group rules according to their output port numbers. Then, within each group, we derive common wildcard patterns for the selected address field. We insert patterns in a pattern tree structure formed by a new pattern dominance relation to detect and avoid rule conflicts. At last, we choose the best set of address patterns by a ranking function. On the one hand, using common wildcards provides a better chance of aggregating rules and achieving higher compression ratios, but on the other hand, it considerably increases the complexity of the compression algorithm due to the growth of conflicting rules with different output port numbers. WildMinnie strongly outperforms Minnie when rule sets have a wide diversity of source and destination addresses, and it is guaranteed that the performance of WildMinnie, in the worst case, is reduced to the performance of Minnie.

The main contributions of this paper are summarized as follows:

- To the best of our knowledge, this is the first paper explicitly and thoroughly explores the conflict issues in general wildcard addresses and provides practical solutions to resolve them.
- We define a new pattern dominance relation as a generalized form of the longest prefix relation, which is used in legacy IPv4 networks to resolve conflicts between wildcard patterns with wildcard bits only at their ending.
- We propose a pattern tree structure to speed up conflict detection and resolution based on pattern dominance relation.
- This paper proposes WildMinnie, a new heuristic solution to compress the rule table of an SDN switch. WildMinnie works with general non-prefix wildcard addresses without particular assumption or limitation and provides solutions to detect and resolve conflicts. WildMinnie algorithm focuses only on the compression concept and does not include routing or rule distribution practices.

The rest of the paper is organized as follows: In the next section, we briefly review the research related to the TCAM space limitation issue. In “Network Model and Problem Statement”, we define the network and the problem model. “Minnie Compression Principles” gives a detailed description of the Minnie algorithm and the philosophy behind it. In “Preliminaries” we study issues related to the rules with general wildcard fields and introduce their various conflict types. We also define a new set of operators and relations to detect and resolve the conflicts and derive common patterns. In “Wildminnie Algorithm”, we explain WildMinnie compression algorithm step-by-step. “Simulations” describes our simulation settings and presents the performance results of WildMinnie with different settings. Finally, “Conclusion” concludes the paper.

## Related Work

Compressing rules with prefix-only wildcards has a very old and rich literature in IPv4 networks. To mention a few, we can indicate to one-dimensional solutions (Draves et al., 1999; Rétvári et al., 2013), a two-dimensional approach (Rottenstreich et al., 2013), and multidimensional approaches like TCAM unification framework (Norige, Liu & Torng, 2013) and TCAM Razor (Liu, Meiners & Torng, 2009). Further references could be found in Braun & Menth (2014a).

SDN Rule placement and compression methods are also surveyed in several papers like Rawat & Reddy (2016) and Nguyen et al. (2015b). We review the related researches in four categories. Some researchers distribute all network rules in the table of the switches in the data plane with a rule placement algorithm. When switches receive an unknown packet, they hand it to each other in a pre-planned path, usually employing a default or an aggregated rule until a matching rule is found for the packet inside a table of a switch (Yu et al., 2011; Nakagawa et al., 2013; Kang et al., 2013; Kanizo, Hay & Keslassy, 2013; Sheu, Lin & Chang, 2018). These approaches mainly route some of the flows through non-optimized paths or discard flows if they do not find a suitable path. In fact, in these approaches, the path dictates the rule placement (Assefa & Özkasap, 2019). Compressing methods can be used along with these approaches to make extra room for better rule placement.

Several works suggest having software switches or some slower storage inside switches (Mimidis-Kentis et al., 2018; Katta et al., 2014) to keep a large number of rules in the data plane. They use cache management algorithms to smartly detect the important flows and fetch them to the TCAM beforehand. Such side memories reduce the delay of handling new flows and significantly decrease the controller’s overhead. However, they need a new type of hardware and require special treatment from the controller (Rifai et al., 2017) and also increase the computational complexity due to the rule dependency problem between software and hardware switches (Mimidis-Kentis et al., 2018; Bera, Misra & Jamalipour, 2019). Compression algorithms may be used as a complement for these approaches to provide compressed caching storage.

The next group of studies provide an optimization model of the network and suggest simultaneous routing and aggregating methodologies to distribute rules and satisfy specific characteristics. Officer (Nguyen et al., 2015a) provides a general framework for the rule allocation problem in resource-constrained networks with a relaxing routing policy. However, the relaxation of the routing policy causes the drawback of longer paths. The researches presented in Huang et al. (2015) and Kosugiyama et al. (2017) propose heuristic algorithms to reduce the total number of flows while respecting the end-to-end QoS. Ashraf (2016) and Galan-Jimenez, Polverini & Cianfrani (2018) focus on minimizing the number of update messages through smart rule aggregation. A new line of researches also considers the two crucial but opposing characteristics of a network: the flow visibility problem and the rule compression. To have the best flow visibility, controllers must use exact-match rules, which means installing one rule for each flow in each switch through its path to the destination. On the contrary, for compressing, rules must be aggregated into wildcard rules. Researches in Bera, Misra & Jamalipour (2019), and Zhao et al. (2020) propose a balanced approach to keep a sufficient amount of network visibility while reducing the total number of installed rules. Another compression scheme (Zhang et al., 2018) proposes multiplexing of rules with the same destination by VLAN ID field in the core of the network. This paper reports an average 15.7% compression ratio and needs special SDN switches.

The last group of approaches, more aligned with our proposed method, reduce the number of rows in rule tables by aggregating them according to some factors. BitWeaving (Meiners, Liu & Torng, 2011) attempts to squeeze several policies into fewer rules to reduce the required TCAM memory and is close to our problem formulation and assumptions. Authors convert non-prefix wildcards into a prefix wildcards and use bit-swapping and bit-merging techniques to combine rules with the same decision where they differ in only one bit. BitWeaving search space is limited and reports only a 23.6% compression ratio. A faster version of BitWeaving is also presented in Luo, Yu & Li (2014). Giroire, Moulierac & Phan (2014) consider compression of rules using the default rule only in the context of energy-aware routing. XPath (Hu et al., 2015) is mainly an explicit path control system that aggregates convergent paths. Braun & Menth (2014b) suggests a longest-prefix-based rule compression, which succeeds in getting only a 17% compression ratio. Our method is similar to theirs in using wildcards, but we use general wildcards instead of only prefixes. Minnie compression is introduced in Rifai et al. (2015) and extensively simulated and tested in Rifai et al. (2017). They report Minnie can compress rules up to 99% in all-to-all scenarios that is the best ratio reported among compression methods, to the best of our knowledge. However, as we will explain in “Minnie Compression Principles” and show with simulations in “Simulations”, Minnie is very sensitive to traffic distribution and works only with the non-wildcard rules. Giroire, Havet & Moulierac (2016), which can be considered as the base of the Minnie algorithm, proved that the rule compression problem is NP-hard.

These solutions suffers from the following weaknesses:

- Some approaches fail to compress efficiently in edge nodes due to the diversity of source-destination pairs in edges (Rifai et al., 2017).
- They do not clearly define the aggregation methodology or use only the default rule aggregation, so their compression ratio usually remains around 20% (Giroire, Moulierac & Phan, 2014; Nguyen et al., 2015a; Hu et al., 2015; Zhang et al., 2018).
- Some of the approaches are not capable of handling wildcard or non-prefix addresses (Braun & Menth, 2014b; Rifai et al., 2017; Assefa & Özkasap, 2019).
- Finally, most of the proposed routing methods do not consider rule conflict issues in aggregation (Huang et al., 2015; Kosugiyama et al., 2017; Bera, Misra & Jamalipour, 2019; Zhao et al., 2020).

This paper presents a new compression algorithm, WildMinnie, which works with Minnie’s principle in its heart but uses general wildcard address patterns for grouping and compressing rules. It handles wildcard rules and also detects and resolves all types of rule conflicts. WildMinnie is proposed for aggressive compression in any layer of the network and only focuses on the ruleset of one individual switch and performs compression based on general wildcard pattern laws defined by OpenFlow standard (OpenFlow Switch Specification ver. 1.4.1, 2015). In this regard, the operation of WildMinnie is not tied to any particular routing method and does not consider the path of flow or other properties like QoS for compression. Moreover, since WildMinnie aggregates rules for compression, it inevitably reduces the flow visibility.

## Network Model and Problem Statement

We model an SDN network with a graph *G*(*S*, *E*) in which *S* is a set of SDN-enabled switches, and *E* is the set of edges that connects them. We use symbols *T*_*s*_ and |*T*_*s*_| to refer to the rule table of switch *s* and its current number of rules. We also show the maximum capacity of a rule table with *T*^*max*^. Each flow is known with a tuple (*s*, *t*, *d*) where *s*, *t* ∈ *S*, are the source and destination, and *d* ∈ *R*^+^ is the load of the flow. To route packets of a flow, a rule is installed in all switches of the flow path toward the destination. Each rule in the rule table is shown by (*s*, *t*, *p*, *L*) in which *s*, *t* are the source and destination addresses, *p* is the output port number, and *L* is the precedence of the rule. In OpenFlow standard (OpenFlow Switch Specification ver. 1.4.1, 2015), the default or table-miss rule ( $*$, $*$, *p*, 0) has the lowest precedence which is 0. This rule matches all packets of all flows. Although in OpenFlow, several other packet header fields can be used for matching apart from these four fields, they are mostly not maskable. Since we heavily use masking and wildcards for compression, we do not consider other fields in our model to keep brevity.

Problem Statement: The problem is finding the aggregate-able rules in the given set of rules, ***R***, such that the aggregated rules comply with the dictated routing policy by the controller.

The above problem statement emphasizes that aggregation of rules must not violate the flows’ routing path which means the output port of rules must be preserved.

## Minnie Compression Principles

In this section, we explain the core principles of Minnie compression. To find the best compression ratio, Minnie executes the compression process twice, once based on the source address and the next time based on the rules’ destination address. Due to the high similarity of source-based and destination-based procedures, we only explain the source-based procedure as depicted in Algorithm 1. Minnie starts with grouping a set of rules based on their source addresses. (*i.e*. ${G_s} = \{ {G_{{s_1}}},{G_{{s_2}}},...,{G_{{s_n}}}\} ,{G_{{s_i}}} = \{ (s,t,p,l)|s = {s_i}\}$, assuming *n* different source addresses). Since all rules in a group have an equal source address, each rule belongs to only one group (*i.e*. $\forall$*i*,*j ≤ n*, *Gs*_*i*_ ∩ *Gs*_*j*_ = $\emptyset$). In the next step, Minnie finds the most frequently used output port in each group and among all groups, denoted by $p_{{s_i}}^*$ and ${p^*}$, respectively.

**Algorithm 1:** Minnie source-based compression procedure based on Rifai et al. (2017).

1: procedureMinnie(*R*, *T*) #*R*: set of rules
#*T*: compressed rules
2: *C*_*r*_: {} # list of rules
3: for each *s* ∈ *V* do
4: $\rm {\bf P_s}^*$ := set of most occurring ports *p* in {(*s*,*t*,*p*)| $\forall$*t* ∈ *V*}
5: ${p^*}$ := most occurring port in all $\rm {\bf P_s}^*$
6: end for
7: for *s* ∈ *V* do
8: if ${p^*}$ ∈ $\rm {\bf P_s}^*$ then
9: ${p_s^*}$ := ${p^*}$
10: else
11: ${p_s^*}$ := most occurring port in $\rm {\bf P_s}^*$
12: end if
13: end for
14: for (*s*,*t*,*p*) ∈ *R* do
15: if *p* ≠ ${p_s^*}$ then
16: ${C_r} \cup = (s,t,p)$
17: end if
18: end for
19: for *s* ∈ *V* do
20: if *p* ≠ ${p^*}$ then
21: ${C_r} \cup = (s,*,p_s^*)$
22: end if
23: enf for
24: ${C_r} \cup = (*,*,{p^*})$
25: end procedure

$\forall ({s_i},{t_j},{p_j},{l_j}) \in {G_{{s_i}}},{C_i}(p) = \sum\limits_{{G_{{s_i}}}} \{ ({s_i},{t_j},{p_j},{l_j})|{p_j} = p\}$

$p_{{s_i}}^* = {\rm{arg}}\;\mathop {{\rm{max}}}\limits_p {\rm{ }}{C_i}(p)$

${p^*} = {\rm arg{\kern 1pt}\; max}\; C(p)$

Finally, all rules with ${p^*}$ output port are replaced with the default rule ( $*$, $*$, ${p^*}$, 0) and all rules with $p_{{s_i}}^* \ne {p^*}$ are substituted with aggregate rules like $({s_i},*,p_{{s_i}}^*,1)$. Other rules within each group with different output ports than the most-used output ports are copied to the table with priorities higher than 1.

The optimum compression ratio is obtained if $\forall$*i* ∈ [0,*n*], $\forall$(*s*_*i*_, *t*_*j*_, *p*_*j*_, *l*_*j*_), *p*_*j*_ = ${p^*}$, which means in all rules output port number is equal to ${p^*}$ and they can be replaced with only one default rule. Of course, there is no need for a switch in such a condition.

The best compression ratio in a rather realizable scenario will be achieved if all rules in a group have an equal output port (*i.e*. $\forall ({s_i},{t_j},{p_j},{l_j}) \in {G_{{s_i}}},{p_j} = p_{{s_i}}^*$). In this condition, all rule groups reduce to only one aggregate rule of the form $({s_i},*,p_{{s_i}}^*,1)$ and the total number of rules will be the number of groups that directly is related to the number of different source addresses.

### Minnie limitations

It is not hard to conclude that Minnie fails to compress when there is only one flow for each pair of source and destination addresses. In this regard, Minnie is very traffic sensitive and has efficient compression when flows are initiated from limited sources or destined to a limited set of destinations.

**Figure 2:**
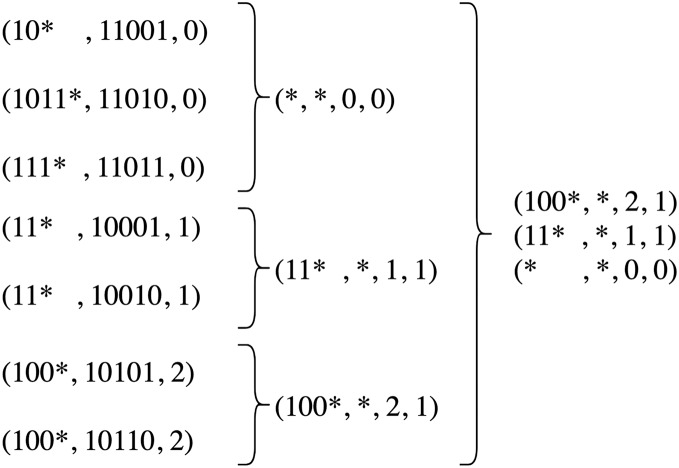
An example rule table and its compressed form resulted from source-address-based Minnie compression.

Another serious limitation of Minnie is the inability to handle wildcard rules since it works based on exact equality of addresses and has no conflict resolution mechanism. As an example, consider a rule table shown in Fig. 2. In this rule table ${p^*}$ = 0, $p_{11*}^* = 1$ and $p_{100*}^* = 2$. Consequently, all rules with the output port of 0 are replaced with the default rule, and the other two groups are reduced to two rules. Now consider a packet with the source address of *s* = 11,100. This packet matches with (111 $*$, $*$, 0) rule in the uncompressed table, while in the compressed table matches with (11 $*$, $*$, 1, 1) rule. This happens because Minnie does not consider the conflict of rule spaces when addresses are in the wildcard form. According to this limitation, Minnie obligates using only non-wildcard, host addresses which means subnet addresses could not be used in rule tables, whereas using subnet addresses is very common, especially in the network core.

Our suggested solution is also based on the same grouping philosophy used in Minnie; however, instead of using exact values of addresses, we drive and use wildcard patterns for grouping, which helps put more rules in one group and get more compression ratio. Besides, using wildcard patterns makes compression performance noticeably independent of the traffic pattern.

## Preliminaries

The main idea in WildMinnie is to group the rules with wildcard patterns of the addresses having the same output port. Like Minnie, an aggregate rule is inserted into the table instead of all rules of a group. We are interested in minimizing the number of patterns to minimize the final number of rules. However, to minimize the number of patterns, we have to use more general patterns with the high count of wildcard bits, increasing the conflict with other rules having different output port numbers. Rule space conflicts severely reduce the final compression ratio. The most critical part of WildMinnie deals with this trade-off by detecting and avoiding the conflicts. Before describing the algorithm of WildMinnie, the following section reviews the specification of the wildcard address format defined in OpenFlow standard and the issues related to their usage.

### OpenFlow wildcard rules

OpenFlow standard, like IPv4, allows expressing source and destination address of rules in the form of wildcards. Wildcard patterns make it possible defining of aggregate rules that match with several flows. In IPv4 standard (Fuller et al., 2008; Rekhter & Li, 1993), wildcard rules could only be in the prefix form (*i.e*. 0111 $*$). With this standard, we can define aggregate rules for flows with a common sequence of 0s and 1s at the start of their source or destination addresses. Usually, the length of the prefix is shown in pattern/len form, like 0111 $*$/4.

Wildcard definition in the OpenFlow Switch Specification ver. 1.4.1 (2015) is more general and allows wildcard bits to be defined in any number and anywhere in the address. For example, 011?101??0 $*$ pattern includes all addresses beginning with 011, then any 0 or 1 bits, followed by 101 bit sequence, then have two other wildcard bits lead to a 0 bit. ‘ $^*$’ indicates that the address’s remaining bits do not matter and can be any sequence of 0s and 1s. Naturally, more complicated patterns can be defined according to this scheme compared with prefix-only wildcards.

Generally, wildcard patterns may have rule conflicts or overlaps; that means it is possible an address matches with several wildcard patterns. Assuming 6-bit addresses for simplicity and having three rules: (110110, $*$, 6), (110100, $*$, 6) and (111100, $*$, 5), all three source addresses match with the wildcard pattern 11?1?0 while they have different output ports. When facing such conflicts, two questions should be addressed: which rules are the best set to be aggregated using the specified wildcard pattern, and how to place the other rules in the table to preserve the original forwarding policy.

For the first issue, we use the strategy of Minnie. The matching rules with the output port having the highest count are aggregated by the wildcard pattern to gain the best compression ratio.

The precedence of rules solves the second issue. In legacy IPv4 routers, the precedence of rules is determined implicitly by the Longest Prefix Matching (LPM) law. According to the LPM, if a packet matches several wildcard patterns, it will be forwarded using the rule that has the longest prefix. In the OpenFlow standard, it is possible to assign a 16-bit precedence value for each rule. Although OpenFlow does not explicitly specify any precedence assignment mechanism, it is possible to implement almost any precedence assignment system in the network controller. For instance, to have an LPM mechanism, it is sufficient to assign the precedence of rules according to the length of their source or destination wildcard prefix. The condition in which a packet matches several rules with the same precedence but different output ports is undefined in OpenFlow specification.

According to these explanations, we can compress the three example rules as: (110100, $*$, 5, 2) and (11?1?0, $*$, 6, 1). The more specific the address pattern, the higher the precedence. Thus, it is forwarded with the highest precedence rule (110100, $*$, 5, 2) rule when a packet matches both rules.

Using general wildcards creates more complicated conflict types like (11?1?0, $*$, 5, 1), (111??0, $*$, 5, 1), and (1111??, $*$, 6, 1), (11?00?1, $*$, 6, 1) where the old LPM mechanism does not help us to determine which pattern is more specific than the other. This necessitates defining a new set of operators and relations for wildcard patterns to help us assign priorities correctly.

### Definitions and operators

We define ${\bf B}^{*}$ system in which variables can have three values of {0, 1, $*$}. A $*$ bit indicates that bit may have any of 0 and 1 values. Pure and wildcard addresses, shown by upper case letters, are vectors of ${\bf B}^{*}$ bits: $\forall$*i* ∈ [0,*k*], *a*_*i*_ ∈ ${\bf B}^{*}$ *A* = [*a*_*k*_, …,*a*_1_, *a*_0_]. We define Mask operator (⊛) as follows:

(1) $$\forall i,j,k \in {{\bf B}^{\ast}},k = i \circledast j = \left\{ {\matrix{ {1,} & {i = j} \cr \ast, & {i \ne j} \cr } } \right.$$

The mask vector always includes sequence of 1s and $*$s. The common wildcard is obtained by applying a mask vector to a wildcard address using Derivation op( ${\rm \odot }$):

(2) $$\forall i,m,k \in {{\bf B}^{\ast}},k = i{\rm \odot }m = \left\{ {\matrix{ {i,} & {m = 1} \cr \ast, & {m = \ast} \cr } } \right.$$

Two bits in ${\bf B}^{*}$ match if both bits are the same or one of the bits is $*$.

(3) $$\forall a,b \in {{\bf B}^{\bf *}},a \approx b\;{\rm if}(a = b) \vee (a = *) \vee (b = *)$$

We say an address *A* matches with a wildcard pattern *W* if they match bit by bit:

(4) $$A \approx W = \mathop \forall \limits_{i = 0}^k {a_i} \approx {w_i}$$

A wildcard pattern *W* is dominant ( $\succ$) of *A* if:

(5) $$W \succ A\; {\rm if}\mathop \forall \limits_{i = 0}^k {w_i} = {a_i} \vee {w_i} = *$$

Based on the dominance relation, the parent pattern always has more $*$ bits than the child, and therefore, has more matching addresses. In contrast, the child pattern is more specific and matches with a limited set of addresses. As a result, if **A**_**c**_ and **A**_**p**_ are the set of addresses that match with the patterns *W*_*c*_ and *W*_*p*_, respectively, and ${W_p} \succ {W_c} \Rightarrow {{\bf A}_{\bf c}} \subset {{\bf A}_{\bf p}}$.

Dominance relation can be considered as a generalization for the longest prefix in which wildcard bits can be anywhere, not just at the pattern’s tail. Employing this relation, we will build a tree structure for wildcard patterns to detect and solve conflict problems.

## Wildminnie Algorithm

WildMinnie compresses rules in three steps as shown in Algorithm 2. In the first step, WildMinnie finds common wildcard patterns. In the next step, WildMinnie puts patterns in a tree-shaped data structure called pattern tree. This structure helps WildMinnie to trim and rank patterns and also detect and avoid conflicts. In the third phase, WildMinnie iteratively selects the best patterns and assigns their priorities based on two factors: the position of a rule in the pattern tree and its ranking. In the following subsections, we explain each step in more detail.

**Algorithm 2:** WildMinnie main procedure and finding common patterns function.

1: procedure wildminnie(*R*, *T*_*s*_) #*R*: set of rules
#*T*_*s*_: rule table of switch *s*
2: *W* := find_common_patterns(*R*) #Step 1, finding patterns
3: *B*_*s*_ := build_pattern_tree(*W*) #Step 2, processing patterns
4: copy_to_switch_table(*B*_*s*_, *T*_*s*_) #Step 3, Writing prioritized rules to the switch table
5: end procedure
6: function find_common_patterns(*R*)
7: *W* : {} #wildcard pattern set
8: *G*_*p*_ := group_rules_by_outport(*R*)
9: for each *Gp*_*i*_ ∈ *G*_*p*_ do
10: $L: = \cup _{j = 0}^{|{G_{{p_i}}}|}({G_{{p_i},j}},{p_i},\{ {G_{{p_i},j}}\} )$ #*L*(*A*,*p*,*M*): a set of tuples
11: for REPEAT_COUNT do
12: for *j* := 0 *to* |*L*| − 2 do
13: *A* :*= L*[*j*].*A* ${\rm \odot }$ (*L*[*j*].*A* ⊛ *L*[*j*+1].*A*)
14: *M* :*= L*[*j*].*M* ∪ *L*[*j*+1].*M*
15: $L \; \cup = \{ (A,L[j].p,M)\}$
16: end for
17: shuffle_set(*L*)
18: end for
19: $W \; \cup = L$
20: end for
21: return *W*
22: end function

### Step 1: finding initial common wildcard patterns

In the first step, WildMinnie finds common wildcard patterns between addresses using ⊛ (Eq. (1)) and ${\rm \odot }$ (Eq. (2)) operators shown in lines 6–22 of Algorithm 2. As final patterns are formed by the output port number, it searches for common patterns among rules with equal output ports. Therefore, in the beginning, all rules are grouped by their output ports. Assuming *m* ports for a switch, we have:

(6) $${G_p} = \{ {G_{{p_1}}},{G_{{p_2}}},\ldots ,{G_{{p_m}}}\}\ {\rm where}\ {G_{{p_i}}} = \{ (s,t,p,l)|p = {p_i}\}$$

The group with the most rule count is handled by one default rule and needs no further processing. For the remaining groups, to get the best compression ratio, we should find a set of patterns that collectively match all initial addresses, while each pattern should have the minimum conflict with patterns of other groups.

Finding all common patterns needs checking of all address pairs, which is in the order of *O*(|*Gp*_*i*_|^2^). For addresses of length 32 bits, this exhaustive process may produce 3^32^ patterns. In an experiment, about 4 million unique patterns were found for only 4,000 rules. Exhaustive searching for all patterns and optimizing the large quantity is unacceptable from both processing and timing aspects.

As an approximation, we derive the common patterns from the adjacent rule pairs in the rule list. Then, the combined list of initial and new patterns is shuffled, and the derivation process is done again. This process is repeated REPEAT_COUNT times which makes this step of order *O*(*Gp*_*i*_). In our experiments, we set this parameter to 2. Although the random approach may not generate the optimum set of patterns, we observed that throughout the WildMinne procedure, a considerable percent of patterns are removed due to low rank, and new high-ranked patterns take precedence. Additionally, more repetition of this process often generates patterns with many star-bits having higher conflicts and minor ranks such that they do not affect the final pattern list. In this regard, the starting random phase provides only a limited set of patterns as initial seed, since new high-ranked patterns are generated during the second step of WildMinnie in a more targeted way. In “Simulations”, we investigate the effect of more repeat counts by a set of simulations.

Initial and derived wildcard patterns are saved as a tuple of (*A*, *p*, *M*) where the first element is the pattern, the second element is the output port number, and the last element is the set of merged initial patterns. The initial pattern set is the set of distinct addresses obtained from the ruleset, which is Minnie’s output, too. The central part of the WildMinnie algorithm operates on this set instead of individual rules. When a pair of patterns are combined and generate a new pattern, initial matching patterns are saved along with the new pattern. This practice accelerates the rules’ processing and helps WildMinnie consider the initial patterns only once. Intuitively, the third element of all initial addresses has only one member, which is the same as the first element (see Algorithm 2 line 10). All derived patterns from all output ports are saved in a global list of pattern tuples (see Algorithm 2 line 21).

**Figure 3:**
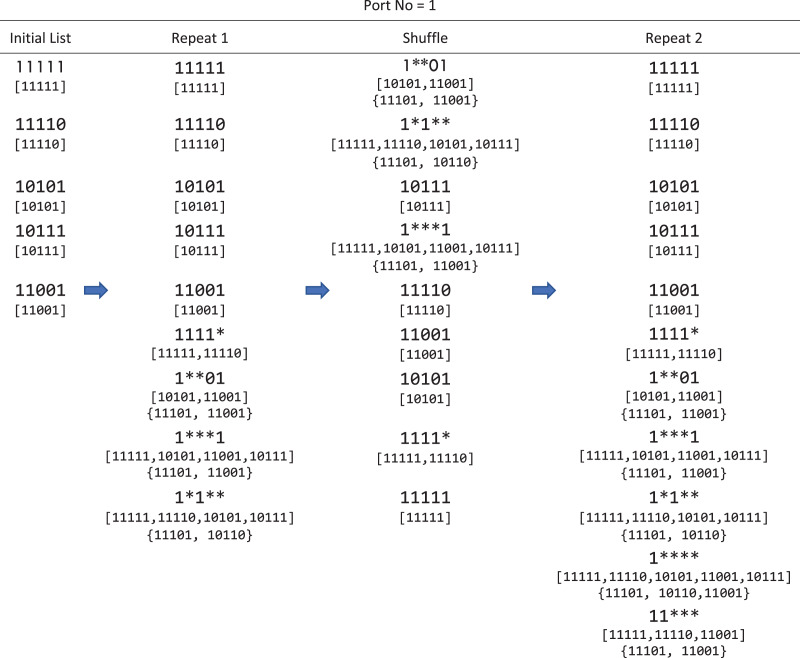
An example of finding common patterns in WildMinnie with two repeats for an initial list of patterns [11111, 11110, 10101, 10111, 11001] having output port = 1. This example assumes another set of initial patterns with output port = 2 as [11101, 11001, 10110]. The list in brackets [] shows the merge set (*k.M*) and the list inside {} indicates the conflicting list (*k.V*) of a pattern.

Figure 3 illustrates the steps of finding common patterns for a group of rules with output port number 1. In this example, for completeness and showing conflicts, we also assume a group of rules with output port number 2. In each step, the merge set and conflict set of each pattern are presented under the pattern.

### Step 2: building pattern tree

In the second step, WildMinnie builds a tree structure using patterns and ranks them. The pseudo-code of this step is shown in Algorithm 3. To compute rank and build the tree, WildMinnie processes all patterns to achieve more information about them. This is done at line 4. After processing, a three-element pattern tuple, *w*(*A*, *p*, *M*), is expanded into a seven-element tuple, *K*(*A*, *p*, *M*, *I*, *V*, *S*, *r*). *K.I* is the set of rules matching the pattern and having the same port as *K.p*. *K.V* is the set of conflicting rules that match *K.A* but have a different port number than *K.p*. The last two elements *K.S* and *K.r* are the set of sub-patterns and pattern rank, respectively. At this step, *K.S* is empty and *K.r* = 0. We will explain these two parameters later.

**Algorithm 3:** Step 2 of WildMinnie.

1: function build_pattern_tree(*R*,*W*)
2: *B: PatternTree*
3: for each *w*(*A*, *p*, *M*) ∈ *W* to
4: *k*(*A*, *p*, *M*, *I*, *V*, *S*, *r*) := process(*w*, *R*)
5: if *k.V* = *ϕ* then
6: *k.r* := compute_rank(k)
7: Insert_Tree(*B*,*k*)
8: else if *k*. *S* $\subset$ B then
9: Insert_Tree(*B*,*k*)
10: choose_best(*k*)
11: *k.r* := compute_rank(k)
12: else
13: *k.S* := find_common_patterns(*k.V*)
14: *W* ∪ = *k.S*
15: end if
16: end for
17: return *B*
18: end function
19: function compute_rank(*k*)
20: $gen := 1 + {\mathop \sum \limits_{s \in k.S}}\left( s.|V| * C+ \sum_{q \in s.S} {\rm compute\_rank}(q)\right)$
21: return $\frac{|k.I|}{gen}$
22: end function

If a pattern has no conflicting rules (*i.e. k.V* = *ϕ*), its rank can be computed and it is also eligible to be inserted in the pattern tree (lines 5–7). Such patterns substitute all of their *K.I* set in the table. As we will explain, a pattern without conflicting rules is a leaf node.

If a pattern has conflicting rules, it can also replace all rules in *K.I*, but its conflicting rule set can not be compressed and must be written as-is to the switch table, adversely affecting the compression ratio. To decrease the number of generated rules, WildMinnie recursively finds common patterns between conflicting rule sets. The resulting sub-patterns are saved in *K.S* of the pattern tuple. Each sub-pattern substitutes a set of rules in the conflicting set (*K.V*), which results in a more compressed rule table. In the pattern tree, sub-patterns of *K.S* are inserted as the children of the initial pattern. Sub-patterns are not necessarily tied to their resulting patterns and are processed independently (lines 12–14). As we will discuss in “Step 3. Assigning Priorities”, the final set of patterns are selected based on a ranking function (Wildcard Pattern Ranking). Therefore, when a child node’s rank is higher than its parent, it is used alone in the final list of rules. However, if a parent pattern in the tree has a better rank than its children, all children must be written in the final rule list to resolve conflicts.

This recursive step to find sub-patterns is, in fact, a part of finding patterns, but instead of discovering the whole pattern set at the beginning, WildMinnie starts with an initial pattern seed and seeks for other patterns in a more targeted way. As another advantage, we should note that processing and selecting the best sub-patterns is done for a limited set of children of a pattern, and unselected ones are trimmed at that level. If the whole pattern set is discovered and found in the first phase, the memory footprint can be very high.

A pattern is ready to be ranked and inserted into the pattern tree if all of its sub-patterns are in the tree and ranked (line 8). At this stage, a pattern may have several overlapping sub-patterns. To accurately compute the rank of a pattern, the best set of its non-overlapping sub-patterns is selected based on their rank.

In the subsequent sections, we explain the details of the pattern tree data structure and ranking function. Since choose_best procedure is very similar to the Algorithm 5, it is not listed separately.

#### Pattern tree

For the correct operation of WildMinnie, we have to handle the rule conflicts. Three types of conflicts may occur when using general wildcard patterns. The first type of conflict is when similar patterns are found in different groups having different port numbers. For instance, 1 $*$ $*$ $*$ $*$ $*$ pattern is found in both groups of *Gp*_1_ and *Gp*_2_. In this case, the group with the largest rule count is selected for compression, and the other rules are used intact or may be compressed and saved in the sub-pattern set. In this way, the conflict is removed.

The second type of conflict occurs when addresses match with several patterns in different groups. As an instance, address 111000 matches with patterns 1 $*$ $*$ $*$ $*$ $*$ in *Gp*_1_ and $*$11 $*$ $*$0 in *Gp*_2_. We resolve this type of conflict with a precise precedence assignment procedure, explained in “Step 3. Assigning Priorities”.

The last type of conflict occurs when a pattern is a parent of other patterns with different outport numbers—as an instance, having two rules (101 $*$ $*$, $*$, 3) and (10 $*$ $*$ $*$, $*$, 2), a packet with the source address 10100 matches both rules. The detection and priority assignment of such conflicts in legacy IPv4 networks are far more straightforward than OpenFlow due to prefix-only wildcard patterns and the LPM mechanism. To resolve the third type of conflict, we introduce the Pattern Tree data structure. A Pattern tree, essentially, is an m-ary tree in which parent nodes have dominance relation ( $\succ$, Eq. (5)) with their children. The root of the tree is the all-star pattern. Sibling nodes in a pattern tree have no particular relation but may have conflicting rule sets.

**Figure 4:**
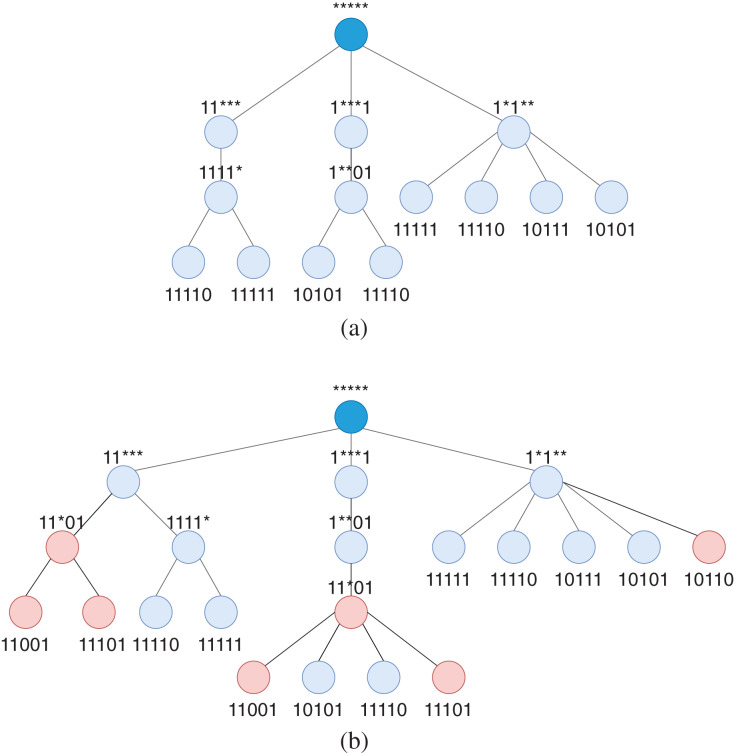
Pattern tree of pattern tuples computed in Fig. 3 with outport of 1, before and after addition of tuples with outport of 2. The root node with the default all-star pattern has a different outport number and has no conflict with the current patterns of the tree. (A) Pattern tree of pattern tuples with *k.p* = 1 after finding common patterns. (B) Pattern tree of pattern tuples with *k.p* = 1 and *k.p* = 2 after finding patterns for tuples with *k.p* = 2. Tuples with outport number 2 are colored in red.

Figure 4A shows the pattern tree for the table of patterns in Fig. 3. In this example, we assume the rules are compressed with the all-star pattern and inserted as the root node has port number 3 and has no conflict with the current patterns. Figure 4B shows the same pattern tree when patterns of rule group with outport of 2 is added to the tree after finding their common patterns. These nodes are colored in red. As it is clear from the figure, conflicts of patterns, especially with different outport numbers, are easily detected in this structure. The pattern tree structure also considerably simplifies the computation and update of patterns’ rank in the last phase of WildMinnie (Step 3. Assigning Priorities) since the rank of a pattern (Wildcard Pattern Ranking) strongly depends on its sub-patterns.

Algorithm 4 shows the procedure of inserting a new node in the pattern tree. Insert_tree encapsulates a pattern tuple in a tree node structure and starts searching a proper position for the new node from the tree root by calling the add_pattern. The add_pattern is a recursive function. In each recursion, it moves one level toward the leaves. In the first step, it is checked if the given pattern can be a parent for some of the current node’s children. If this condition is met, the new node is inserted as a child of the current node (line 10), and all matching children are removed from the current node and added as children of the new node. Otherwise, it is checked if any child of the current node can be a parent for the new node. In this condition, add_pattern function is called for every eligible child, which causes a pattern to be added as a child of one or several nodes (line 16). The last phase of WildMinnie ensures only one of these redundant nodes is used in the final rule list of the switch table (Step 3. Assigning Priorities). When the new node can neither be a child nor a parent for any of the current node’s children, it is appended as a child of the current node (line 18).

**Algorithm 4:** Inserting a pattern in a pattern tree.

1: procedure Insert_Tree(*B, k*)
2: if *k.A* is *ALL_STAR* then
3: return
4: end if
5: *n* := *node*(*k*)
6: add_pattern(*B.root, n*)
7: end procedure
8: procedure add_pattern(*b, n*)
9: $Y := \{y|y \in b.S \wedge n.A \succ y.A\}$
10: if *Y* ≠ $\emptyset$ then
11: *n.parent* := *b*
12: *b.S −=Y*
13: $\mathop {\forall} \limits_{y \in Y}y.parent := n$
14: else
15: $Z := \{z|z \in b.S \wedge z.A \succ n.A\}$
16: if Z ≠ $\emptyset$ then
17: $\mathop {\forall} \limits_{z \in Z}$ add_pattern(*z, n*)
18: else
19: *b.S* ∪ *= n*
20: *n.parent := b*
21: end if
22: end if
23: end procedure

In the worst case, the height of a pattern tree can be as much as the address length when all possible patterns are available, or patterns are linearly the child of each other. Since WildMinnie deliberately keeps a limited number of patterns, the worst-case condition barely happens in practice. Even with more than 200 K flows in our simulations, tree height does not exceed more than six levels. Therefore, the insertion or deletion operations on the pattern tree are efficient regardless of the rules or the selected patterns count in practice.

#### Wildcard pattern ranking

The rank of a pattern is directly related to the number of rules it compresses and the number of rules it generates. The ranking function is implemented in compute_rank function shown in Algorithm 3 at line 19. This function, at first, computes the count of the rules a pattern generates (line 20). Each pattern inevitably generates at least one aggregated rule in replacement of its *K.I* set. As stated before, all of the rules in *K.V* set must be inserted into the table without change. If a pattern has any sub-patterns, they are also used in conjunction with the root pattern. The next part of the formula in line 20 recursively computes the number of rules generated by the sub-patterns. To avoid using patterns with a large conflicting set, we multiply the number of conflicting rules with a large constant (*C*). The rank of a pattern is obtained by dividing the number of replaced rules (|*K.I*|) by the generated rules. The higher rank value indicates the pattern substitutes a larger number of rules with lesser aggregated rules.

### Step 3: assigning priorities

In the last step of WildMinnie, presented in Algorithm 5, rules of the pattern tree are written in the rule table of the switch in their rank order. Procedure copy_to_switch_table in a loop selects the highest rank pattern tuple from the tree. If the output port is the same as the most frequently used port number, it is discarded, as all of these rules will be replaced by the default rule. Then, wildcard patterns of the selected tuples are copied into the switch table, recursively using the add_rules procedure (line 14). Inside this procedure, sub-patterns are added to the table, first with higher priority, and then the node’s pattern is added. The priority of rules is decremented by the addition of each rule to prevent the second type of conflicts as explained in “Pattern Tree”.

**Algorithm 5:** Assigning precedence to rules.

1: procedure copy_to_switch_table(*B, Ts*)
2: *priority* := *HIGHEST_PRIORITY*
3: *M* : {}
4: while *B* ≠ $\emptyset$ do
5: *b* := highest_rank_node(*B*)
6: if *b.p = ${p^*}$* then
7: continue
8: else if *b.M* $\subset$ *M* then
9: continue
10: end if
11: add_rules(*b*,*T*_*s*_, *priority*,*M*)
12: end while
13: end procedure
14: procedure add_rules(*b*, *T_s_*, *priority*, *M*)
15: $\forall$_*s∈b.S*_ add_rules(*s*, *T_s_*, *priority*)
16: add_rule(*T*_*s*_, (*b.A*, * $*$*, *b.p*, *priority*))
17: *priority* −= 1
18: *M* ∪*= b.M*
19: end procedure

All initial patterns in *k.M* of the copied pattern are marked (line 18) as covered. Having a coverage log helps WildMinnie to discard patterns that have a high rank, but their merging set has been covered previously (line 8).

### WildMinnie analysis

WildMinnie finds common patterns in the first step. If we want to visit each address once, the time complexity of finding patterns will be from the order of *O*(|*T*_*s*_|). If |*T*_*s*_| unique patterns are found, their insertion in the pattern tree will be from the order of *O*(|*T*_*s*_|^2^). Similarly, the last step of WildMinne involves sorting and searching the handled merge set, which is in the same order. Of course, the worst-case scenarios are absolutely rare cases and may not happen in practice. The following theorem defines a lower bound for the compression ratio of WildMinnie.

**Theorem 1** WildMinnie in the worst case performs as Minnie.

Proof. WildMinnie, as illustrated in Algorithm 2, for each pattern keeps a merge list. The merge set have a set of patterns that are directly obtained from the ruleset without masking and derivation process. In this way, the initial patterns set are the same as the final grouping of Minnie. During masking and derivation process in procedure find_common_patterns (Algorithm 2 line 6), WildMinnie keeps track of initial pattern set, too. Therefore, for each pattern generated in any step of the algorithm, it is clear which initial patterns formed that pattern. We also should note that, in the last step of WildMinnie, in Algorithm 5 a pattern is discarded when all of its merge set members are covered by previous patterns. By the above explanation, we can conclude that each initial pattern is used at most once in the final rule set copied to the table. An initial pattern may be used in the original form or merged with other patterns and form a new pattern.

Now, suppose that for a rule set, Minnie compression ratio is better than WildMinnie. This necessitates that some initial patterns are used more than once in various derived or non-derived forms, which contradicts the WildMinnie algorithm.

### Incremental updates

It should be noted that after the network initial start-up, generally, rules will be added to the network in small batches. If the address field of a new rule matches an existing wildcard rule and their output port is also equal, then no change is required in the rule table. Otherwise, a new non-wildcard rule should be inserted in the table with the highest precedence. For a not-too-large set of rules, this approach is fast and has zero overhead. As the WildMinnie compression ratio is good enough, one should not be concerned about several hundreds of new rules. However, the compression ratio will be decreased over time by using this approach.

The alternative approach runs the complete WildMinnie procedure for the limited set of rules that did not match the existing rules. We should keep a pattern tree for each switch and apply incremental updates on it in this method. For incremental deletions, we check the pattern tree’s corresponding nodes for the set of rules intended for deletion. All nodes and their corresponding rules with the condition |*n.I*| = 0 are removed from the tree and the rule table.

## Simulations

In this section, we first explain our simulation settings and then show the performance of WildMinnie using several configurations.

### Simulation settings

We implemented WildMinnie with Java, which is the language for most of the well-known controllers. In simulations, we consider several factors to study the performance of WildMinnie from various aspects. In all simulations, we report the compression ratio on destination address; there was no remarkable difference between the results of sources and destination addresses. We also assume that all flows are active during the simulation time, so switches must have rules of all flows. This assumption is the worst condition for the rule placement.

As WildMinnie does not have an integrated routing method, we use the simple routing method introduced in Minnie, which we call it MinnieRouting. MinnieRouting defines a weight parameter for each link, updated after every rule placement according to the total load of passing flows and the filled ratio of its source switch’s rule table. MinnieRouting distributes flows’ load in the whole network to avoid overloading of some core switches.

#### Topologies

**Figure 5:**
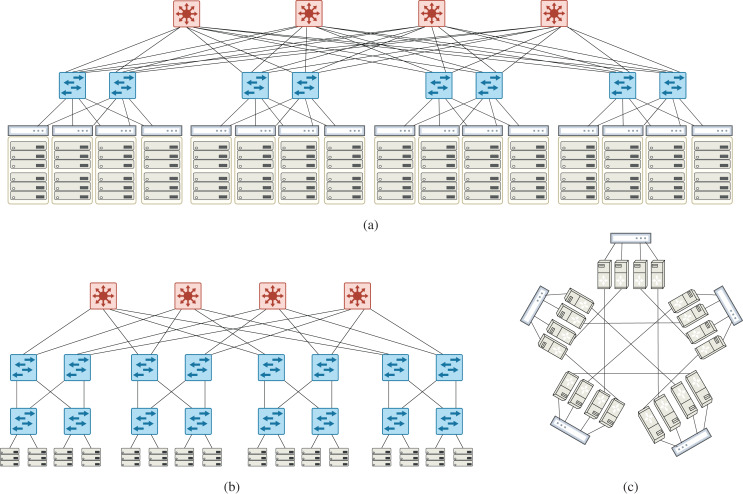
Example of data center topologies used in simulations (A) VL2 topology, *k* = 4 (16 TOR, 8 aggregate, 4 intermediate switches) (B) Fattree topology, *k* = 4 (8 access, 8 aggregation, 4 core switches) (C) DCell(4,1) topology composed of five DCell(4,0).

Similar to Rifai et al. (2017), simulations have been done on well-known data center topologies: Fat-tree (*k* = 4, 8, 12, 16) (Al-Fares, Loukissas & Vahdat, 2008), VL2(*k* = 2, 4, 8, 12) (Greenberg et al., 2009), DCell((2,1), (3,1), (4,1), (5,1), (6,1)) (Guo et al., 2008). These topologies include 9 to 320 switches where each switch has at most 24 ports. With this assumption, some topologies cannot be set up like VL2(*k* = 16). Figure 5 shows an example of these topologies. Fattree and VL2 belong to the family of architectures in which only switches participate in forwarding. In other families of architectures like DCell and BCube, servers also cooperate in forwarding. One purpose of experimenting on various topologies is to produce different mixes of flows to study the compression behavior. Datacenter topologies usually have a standard and efficient count of links and produce balanced mixes of flows. Meanwhile, data centers receive large diversity of source-destination pairs, making them a pretty perfect target for testing compression algorithms. To show the stability of WildMinnie’s performance in extreme scenarios, we also test the shortest path routing instead of MinnieRouting. The general shortest path routing does not balance the flows over links and switches and produces hot points in the network.

#### Test subnet and flow sets

We assume IPv4 addresses with a length of 32 bits. To generate random flows, we produce a determined number of random subnet addresses with prefixes of length 8 up to 32 bits and assign them to the edge nodes by random. In the flow generation process, we rigidly try to use all the subnets assigned to the edge nodes to produce varying pairs of source and destination addresses. Then, we produce different flow sets with 10,000 flows. The number of flows is deliberately chosen to contain enough variety of subnets. We strictly believe that the number of flows in simulations does not transparently reflect the compression performance since one can produce hundreds of flows between a pair of addresses with varying port numbers and achieve a 99.9% compression ratio. Instead of flow count, we consider the following two parameters:

1. **Number of subnets per edge node (SPE):** this parameter determines the minimum number of unique subnet addresses that should be assigned to each edge node. In other words, SPE controls the diversity of source or destination addresses of flows originated or destined to one edge switch. We generate flow sets with SPEs of 1, 10, 20, 40, and 80.
2. **Stickiness**: this parameter determines the probability of using previously used addresses as a source or destination instead of using new ones in the flow generation process. We generate flow sets with three stickiness of 0.25, 0.5, 0.75.

Since Minnie has a severe issue with wildcard addresses, as explained in “Minnie Compression Principles”, all generated flow sets are conflict-free for a fair comparison.

### Performance metrics

For performance comparison, we use two measurements. The first measurement denoted by *C*_*total*_ is defined as follows:

${\rm {\mathbb T}} = \sum\limits_{s \in S} |{T_s}|$

${C_{total}} = 100*\displaystyle{{{{\rm {\mathbb T}}_{old}} - {{\rm {\mathbb T}}_{new}}} \over {{{\rm {\mathbb T}}_{old}}}}$

${\rm {\mathbb T}}$ is the total number of rules installed in all switches of a network and *C*_*total*_ indicates what percent of total rules reduced by a compression method. This parameter is also a good indicator for the average compression ratio of individual switches. The second measurement, *C*_*max*_, shows the effectiveness of a compression method in reducing the maximum size of rule tables.

${{\rm {\mathbb T}}^{max}} = \mathop {MAX}\limits_{s \in S} |{T_s}|$

${C_{max}} = 100*\displaystyle{{{\rm {\mathbb T}}_{old}^{max} - {\rm {\mathbb T}}_{new}^{max}} \over {{\rm {\mathbb T}}_{old}^{max}}}$

A compression method can reduce some rule tables, but it may fail to compress large or special tables. In this condition, average compression may be satisfactory; however, few tables remain with a large count of rules.

### WildMinnie performance

This section provides a performance comparison of WildMinnie and Minnie based on topology, SPE, and stickness parameters.

#### By subnet-per-edge (SPE)

**Figure 6:**
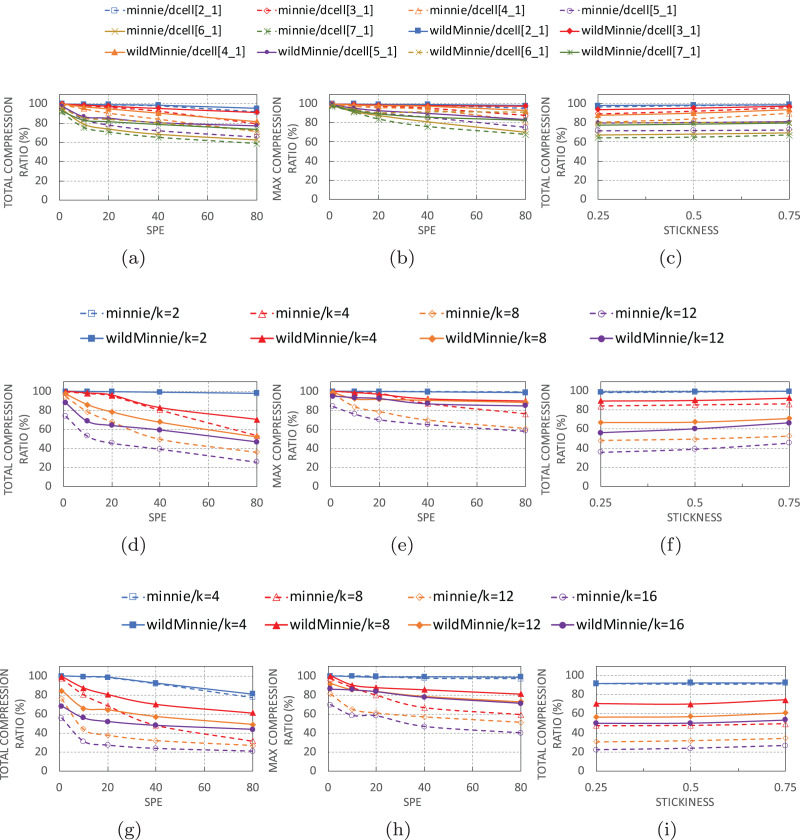
*C_total_* and *C_max_* of WildMinnie and Minne in different topologies with varying SPE and stickiness values. SPE charts use stickiness = 0.5 and Stickiness charts use SPE = 40 (A) *C_total_ vs*. SPE in DCell graphs. (B) *C_max_ vs*. SPE in DCell graphs. (C) *C_total_ vs*. stickiness in DCell graphs. (D) *C_total_ vs*. SPE in VL2 graphs. (E) *C_max_ vs*. SPE in VL2 graphs. (F) *C_total_ vs*. stickiness in VL2 graphs. (G) *C_total_ vs*. SPE in Fattree graphs. (H) *C_max_ vs*. SPE in Fattree graphs. (i) *C_total_ vs*. stickiness in Fattree graphs.

Figures 6A, 6D and 6G  show the performance of algorithms with increasing number of subnets per-edge in thee topologies. In all of these experiments, we use 10K flow sets with stickiness of 0.5.

As expected, the performance of Minnie compression quickly drops with increasing SPE. Especially in VL2 and Fattree topologies, Minnie loses 20–70% of its performance with only 10 subnets per edge. Higher SPEs decrease its compression ratio by less than 30%, but it is not as sharp as the SPE = 10. The sharp beginning drop happens because the first compression ratio is achieved in an unreal condition of having only one subnet-per-edge. In the continue, compression ratio decreases with a reasonably monotone rate of 10–20%. This was pretty predictable since Minnie’s compression strongly depends on equal addresses, and with higher SPE values, the variety of destination addresses destined to the same switches grows.

WildMinnie also loses its compression performance with SPE, but the loss slope is slower than Minnie. The compression ratio gap between Minnie and WildMinnie grows as SPE increases, in a way that in SPE = 80, the compression ratio of WildMinnie is twice Minnie’s approximately.

The compression ratio also decreases when graphs get larger in all types of topologies. This performance loss is natural since the flow set size remains constant while the number of nodes is increased. Therefore, flows are distributed in more paths over a high count of nodes which causes less compression. For instance, in smaller graphs like Fattree *k* = 4, VL2 *k* = 4, and most of the DCELL topologies compression rate is around 99%, since a small number of switches bear a large percent of rules.

These facts are also approved by *C*_*max*_ charts in Figures 6B, 6E, and 6H. According to these figures, WildMinnie, independent of the topology or graph size, successfully reduces the max table size by 80–99%. These statistics confirm that the spread of the rule set in more nodes and smaller table sizes is the main reason for the performance reduction in large graphs. This is in contrast with the behavior of Minnie, which fails to keep the high *C*_*max*_ ratio in large graphs. A diverse set of addresses scattered in more nodes reduces the probability of address equality in switches which again confirms the strong dependency of Minnie to traffic distribution.

#### By flow stickiness

In Figs. 6C, 6F, and 6I, total compression evaluated against stickness parameter with flow sets of size 10 K and SPE of 40. High stickness directly affects the probability of having equal addresses. Increasing stickness from 0.25 to 0.75 improves the compression ratio in both methods by 2–10%. More significant improvements are achieved in larger graphs where nodes have a small set of rules, and equality or similarity of rules has decreased by the load balancing feature of the routing. In this condition, stickness increases the probability of having similar rules. From the architectural view, DCell has the least, and VL2 has the highest percentage of improvement. The reason for this behavior is clarified in the next section.

#### By topology

**Figure 7:**
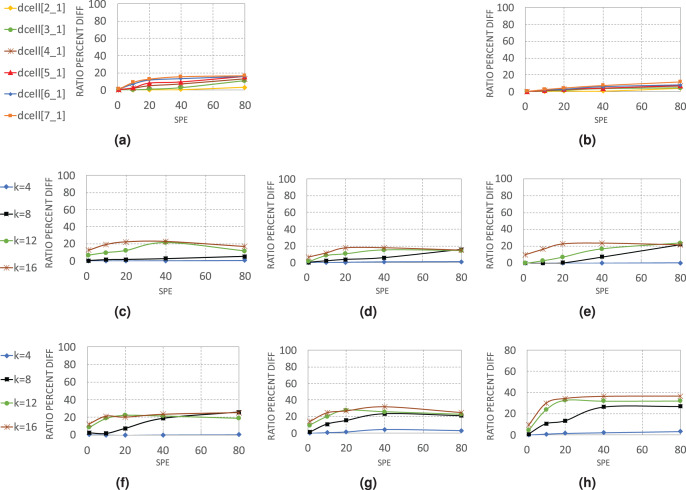
Average of $C^{WildMinnie}_{total} - C^{Minnie}_{total}$ in various layers of data center architectures (A) DCell-edge nodes. (B) DCell-non-edge nodes. (C) VL2-edge nodes. (D) VL2-aggregate nodes. (E) VL2-core nodes. (F) Fattree-edge nodes. (G) Fattree-aggregate nodes. (H) Fattree-core nodes.

Figure 7 gives a detailed view of how WildMinnie and Minnie operate in each layer of architectures. Each chart displays the average difference between the total compression ratios of WildMinnie and Minnie based on different SPE values with a flow set size of 10 K and stickiness of 0.5.

In DCell architecture, since there are no aggregate or core layers, we categorized nodes to edge and non-edge nodes. WildMinnie and Minnie in this architecture have the lowest performance difference, as both of them achieve high compression ratios. In DCell, plenty of paths exists between each pair of nodes, and MinnieRouting performs a good load balance on links and switches. Thus, when the graph size grows, the distribution of rules is not changed significantly. However, with high SPEs, the diversity of addresses increases, and the difference of compression ratio between WildMinnie and Minnie grows slowly.

VL2 and Fattree architectures present a more clear view of Minnie and WildMinnie’s behavior. According to Figs. 7C–7H, the least difference of performance is achieved in edge nodes where the diversity of destination addresses is high, and the most significant performance gap is in the core nodes. In core and aggregate nodes, a good mix of flows with the same destination switch exists; however, WildMinnie gets the most out of this mix by finding the common patterns. In both architectures, graphs of size *k* = 8 show the rising threshold. In the beginning, with SPE = 1, both methods have similar results, but with the growth of SPE and reduction of address equality, WildMinnie takes advantage of its pattern weaving approach and achieves a better compression ratio. VL2, compared with Fattree, has more interconnections between its pods and has better potential for balancing. Therefore, in VL2, the performance gap is lower, and its rising threshold is higher than Fattree.

#### By deeper search

**Figure 8:**
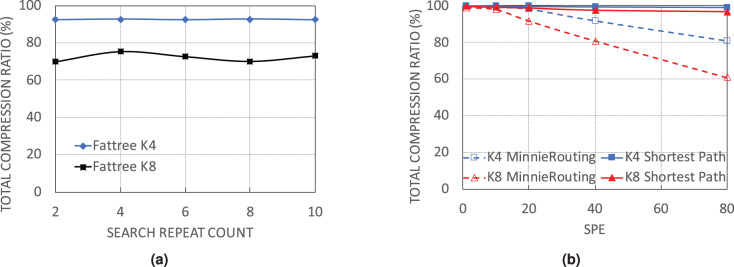
Performance of WildMinnie on FatTree networks with no load balancing and deeper search for finding common patterns. (A) *C_total_* on Fattree networks with different number of rounds for searching of common patterns. (B) *C_total_* with default shortest path routing and no load balance.

The next simulation set examines the performance of WildMinnie with the number of rounds it searches for the common patterns. For testing, the process of combining and shuffling of address patterns is repeated with different values of REPEAT_COUNT parameter in function find_common_patterns (Algorithm 2, line 6). Simulations were carried out with flow sets of size 10K, SPE = 40, and stickness = 0.5 on FatTree architecture as WildMinnie has its least performance on FatTree. We continued the simulations until we received the out-of-memory error. Figure 8A doesn’t show a meaningful relation between the final compression ratio and REPEAT_COUNT. The variation of *C*_*total*_ for this simulation is around 5%, which most probably happens due to the randomness in the process of generating and assigning subnets. We draw two important results from this simulation. First, increasing the search rounds increases the number of patterns exponentially. Second, due to the generation of a large number of low-ranked patterns, an exhaustive search to find a better set of patterns needs a tremendous number of rounds and a huge memory size.

#### By routing method

In this simulation set, we use the simple shortest path routing. With this routing, only one path is always selected between a pair of given source-destination. As a result, most of the switches in the network have a rule table size of zero, while all flows pass through a small set of switches with very large rule tables. In previous experiments, we showed that when rules are distributed on more nodes and switches receive fewer rules, the compression performance of WildMinnie and Minnie is reduced. In contrast, when multiple flows pass a switch, the possibility of finding equal or similar address patterns is increased. With the simple shortest path routing, the second condition occurs in its extreme state. From Fig. 8B we observe the expected behavior from WildMinnie where it successfully compresses rules by more than 99% despite SPE of 80. We did not test Minnie’s performance with the simple shortest path since it is integrated with its routing method.

#### Running time

The execution of WildMinnie includes two main time-consuming parts: finding common patterns and building the pattern tree. For measurement, we choose the FatTree (*K* = 4) network, which has a high rules-per-switch parameter. Measurements have been done on a commodity PC with Intel Core i5-4460 CPU^2^ 2This CPU, released in 2014, has four 3.2 GHz cores with 6M cache. and 8 GB of RAM.

**Figure 9:**
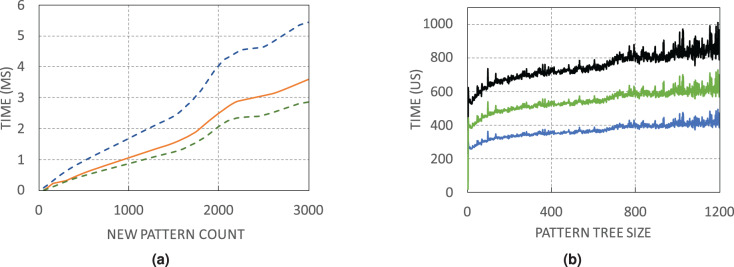
WildMinnie running time measurements (A) Min, average and max time of finding specific number of patterns. (B) Min, average and max time of inserting a pattern into the pattern tree with the specified size.

Figure 9A shows the minimum, average, and maximum running time of finding a certain number of unique patterns. Due to the random selection of address pairs, the number of explored pairs for finding a certain number of unique patterns is not constant. For instance, for 2,000 unique patterns, it may be necessary to visit 25,000 pairs, while in another switch with only 10,000 address pairs, the same count of patterns is found. Search time in WildMinnie with REPEAT_COUNT = 2 increases linearly with a slight slope. According to the Fig. 9A, in the worst case, it takes 3–5 ms by average to find 3,000 unique patterns that are quite a large count in our experiments. The next Fig. 9B shows the minimum, average, and maximum time for processing plus inserting a pattern in the pattern tree with the specified size. By average, it takes about 0.2–1 ms to process and insert a pattern in a tree with 1,200 nodes.

Based on these figures, the total execution time of WildMinnie for one switch takes only several milliseconds on a commodity PC, and it can be used in larger networks with a higher number of flow sets without any concern.

## Conclusion

In this paper, we introduced WildMinnie, a new rule compression algorithm for SDN networks. WildMinnie principally compresses rules by deriving common general wildcards of address fields. We explored rule conflict issues using general non-prefix wildcards defined by OpenFlow standard and introduced solutions for each type of conflict. We tested WildMinnie on well-known data center topologies using flow sets with different source-destination pair diversities controlled by two sub-per-edge and stickiness parameters. We showed that WildMinnie performs better than Minnie, which is the only compression method that claims compression ratios higher than 90%. Especially, WildMinnie is successful in compressing rule sets having large source-destination diversity where Minnie fails to compress effectively.

## Additional Information and Declarations

### Data Availability

The following information was supplied regarding data availability: The source code is available at figshare: Khanmirza, Hamed (2022): WildMinnie Source Code. figshare. Journal contribution. https://doi.org/10.6084/m9.figshare.15131676.v1.
